# Material Properties Influencing the Charge Decay of Electret Filters and their Impact on Filtration Performance

**DOI:** 10.3390/polym12030721

**Published:** 2020-03-24

**Authors:** Jinwook Lee, Jooyoun Kim

**Affiliations:** 1 Department of Textiles, Merchandising and Fashion Design, Seoul National University, Seoul 08826, Korea; shop0319@snu.ac.kr; 2 Research Institute of Human Ecology, Seoul National University, Seoul 08826, Korea

**Keywords:** electret, charge, filtration, humidity, thermal, aging

## Abstract

Electret filters as opposed to mechanical filters display the enhanced ability to capture airborne particles with the electrostatic attraction. However, the environmental aging during shelf-life or use may cancel its benefit by dissipating the charges. This work investigates the polymeric attributes influencing the charge decay and the electrostatic filtration of electret filters, employing polymers with different dielectric constants (ε_r_) and wettability. As accelerated aging, high temperature (120 °C) or high humidity (25 °C, 90% RH) was applied to the electret filters for 48 h. For the humidity aging, wetting property of material was a critical factor affecting the charge decay and the filtration performance, as the absorbed water increases the electrical conductivity. For the thermal aging, the material with the highest ε_r_ deteriorated the electric potential and the filtration performance by the largest extent, due to the lower band gap energy for charge transfer. The results of this study implicate that ε_r_ and wettability are important material parameters influencing the electric conductivity and chain mobility, and they can be used as convenient predictors for charge retention capacity affecting the robust electrostatic filtration performance.

## 1. Introduction

Electrets are charged dielectrics, and they are generally formed through mobilizing the polar groups and trapping the charge carriers at above the glass transition temperature (T_g_) [1,2,3]. In this process, the material in an intense electric field acquires charges, then the charges are frozen at cooling [4]. The electric field in electrets is sourced from the internal polarization of a dielectric or the externally accumulated electric charges [1,4,5,6]. Another simple classification of electrets is hetero-electrets and homo-electrets; where homo-electret mostly occurs by excess charge injection into nonpolar materials, and hetero-electret occurs through the internal charging by orientation of dipoles [3]. Generally, a polymer with high dielectric constant (ε_r_) polarizes more in the electric field, giving a high level of initial charges, while its loss with time is another issue [1,5,7].

Commonly available commercial electret filter media is made by corona-charging. Rather recently, electrospun webs have been regarded as potential electret filter material, while its scalability is yet challenging [8,9,10,11,12]. In most cases, both hetero- and homo-charges exist simultaneously, and the dominant type of charges determine the character of the electret [3]. In practical application, charge retention capacity of electret material is important, as an electret filter exposed to high temperature and humidity during its shelf-life can lose charges with time [10,11]. Moreover, the tendency to respond to such environmental conditions is affected by the material properties. The charge retention capacity is generally dependent on the electrical conductivity and the energy level of localized trap sites [5,13,14,15]. The mechanisms of charge decay are mostly explained by the relaxation of polarized state of dielectrics, spatial distribution of dipoles and free charges, and the neutralizing effect of dielectric conductivity [10,11,16,17,18].

Generally, homo-electrets made from nonpolar materials hold stable charges, due to low electrical conductivity and low water absorption [3,19]. Thus, nonpolar polymers such as polyolefins can be good candidates of electret materials [20,21]. One of the common applications of polymeric electret is air filtering materials. The electret filter as opposed to the mechanical filter display enhanced ability of capturing airborne particles due to the benefit of coulombic attraction and induced polarization. Thus, electret filters tend to give higher quality factor (QF), which is the relative efficiency to a unit pressure drop [6,22,23]. The limitation of electret filter comes from the charge decay, which occurs through environmental aging that causes electric conduction or charge carrier mobility [8,16,17,18]. It has been demonstrated that the exposure of an electret filter to heat, humidity, and solvent can cause deterioration of filtration performance, as a result of charge loss [17,22,24].

The environmental factors that deteriorate the electret filtration have been studied rather frequently [8,17,18,24], probably because it is associated with the practical use conditions; on the other hand, the material aspects that influence the charge decay have rarely been studied. While various material options were examined to attain the high-quality factor filters [10,25,26], it lacked scrutiny on the material attributes that enable the long-term charge retention. Moreover, the dielectric constant (ε_r_) of material has rarely been associated with the charge decay phenomenon in experimental investigation [1,27], while there are implications that ε_r_ is related with the charge stability and conductivity [7,27,28].

**Figure 1:**
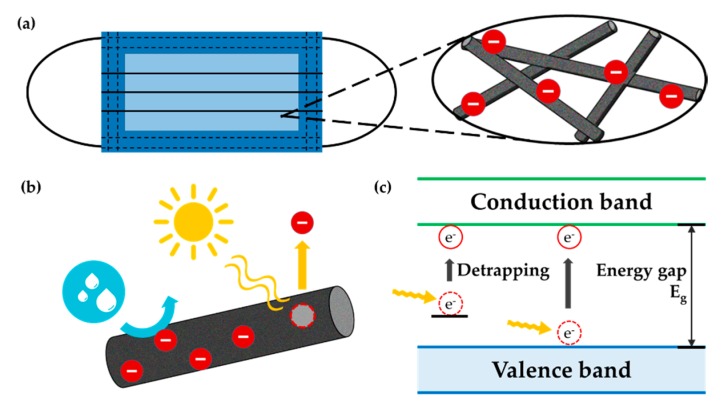
Scheme of study concept. (**a**) Electret fibers applied to a filtering respirator; (**b**) charge decay with the exposure to moisture and heat; (**c**) charge detrapping phenomenon.

The purpose of this work is to investigate the polymeric attributes that influence the charge retention capacity of electret filters. In this study, polymers with three different dielectric constants—polypropylene (PP), polyvinylidene fluoride (PVDF), and polyacrylonitrile (PAN)—were compared for their charge decay phenomena with varied aging conditions. The material properties such as surface energy, moisture regain, ε_r_, and crystallinity were analyzed, and their influence on charge decay and filtration performance were discussed. Ultimately, this study intends to provide informative discussion on the polymeric properties that affect charge retention capability, for the design of robust filter material of which charges sustain longer in harsh environmental conditions. Figure 1 demonstrates the concept of this study.

With the growing environmental concern with the airborne particulate matters (PM), various filter materials are being explored in search of adsorbate specificity, high filtration performance, environmental sustainability, etc. [10,11,22,25]. Most of those studies focus on achieving the high filtration efficiency, but lack the understanding of material properties that make the robust electret filters in consideration of charge retention. This study will broadly impact the robust filter development, by guiding the material selection for the superior electrostatic filtration.

## 2. Materials and Methods

### 2.1. Materials

Polyacrylonitrile (PAN, Mw = 160,000 g·mol^−1^) and polyvinylidene fluoride (PVDF, Mw = 234,000 g·mol^−1^) were purchased from Sigma-Aldrich (Saint louis, MO, USA), and were used to make electrospun filter webs. N, N-dimethylformamide (DMF), and acetone were purchased from Fisher Scientific (Ottawa, ON, Canada) and Daejung Chemicals & Metals Co. (Siheung, Korea), respectively. Two types of commercially available polypropylene meltblown webs were obtained from CHL Korea (Seoul, Korea). Thicknesses of web materials including meltblown and electrospun webs were measured using a thickness gauge (Mitutoyo 7050 Dial Upright Gauge, MITUTOYO, Kawasaki, Japan) within ± 0.01 mm error range.

### 2.2. Electrospinning

A 20 wt.% of PAN prespinning solution was prepared by adding 1.058 g PAN in 10 mL DMF, and stirring for 24 h at 750 rpm and 40 °C. The electrospinning was carried through a 23-gauge needle at the feeding rate of 3 mL/h. The fibers were collected on a drum collector rotating at 100 rpm covered with polypropylene spunbond web, maintaining the tip to collector distance (TCD) to be 12 cm. The applied voltage was set at 15 kV.

For PVDF electrospinning, a 21 wt.% PVDF/DMF/acetone solution was prepared by adding 1.305 g PVDF in 3 mL DMF and 3 mL acetone, then the solution was stirred for 24 h at 850 rpm and 85 °C. The solution was fed through a 21-guage tip, at the feeding rate of 5 mL/h and 22 kV applied voltage. The TCD was set at 12 cm. After the spinning, electrospun web was placed in the hood for 24 h to evaporate the solvent residue.

### 2.3. Accelerated Environmental Aging

As filter media layers, the PAN electrospun web, PVDF electrospun web, and PP meltblown web, respectively, were layered with polypropylene spunbond webs at the top and the bottom. The electric field and the filtration performance of the respective filter media layers were examined before and after aging of filters at different conditions. All samples were conditioned at 20 °C, 65% RH for 24 h before aging. Aging conditions included thermal aging at 120 °C and moisture-aging at 25 °C, 90% RH for 48 h. All conditioning and aging were conducted using a climate chamber (PL-3KPH, ESPEC Corp., Osaka, Japan).

### 2.4. Evaluation of Filtration Performance

The instantaneous filtration performance was examined by a filter tester (TSI 8130, TSI Inc., Shoreview, MN, USA). The test was carried out using NaCl particles in the count median diameter of 0.075 ± 0.02 μm, where the particle charges were neutralized in Boltzmann distribution. The filter media of 40 cm^2^ area was exposed to the NaCl aerosol in an average mass concentration of 25 ± 0.2 g/L. The percentage of particle penetration and the pressure drop of the filter media were examined at the face velocity of 12.5 cm/s (flow rate of 30 LPM). To account the relative efficiency to a unit pressure drop, the quality factor (QF) was calculated as follows.

(1) $$\mathrm{Quality}\;\mathrm{factor}\;\left({\mathrm{Pa}}^{-1}\right)=-\frac{\ln(\%\;\mathrm{penetration}/100\%)}{\mathrm{pressure}\;\mathrm{drop}\;(\mathrm{Pa})}$$

### 2.5. Material Characterization

#### 2.5.1. Moisture Regain

The moisture regains of the conditioned filter media (PAN electrospun web, PVDF electrospun web, PP meltblown web) at 20 °C, 65% RH for 24 h were examined. The weight of the dried sample was obtained by drying the sample at 105 °C for 2 h. Moisture regain was calculated by the following equation.

(2) $$\mathrm{Moisutre}\;\mathrm{regain}\;\left(\%\right)=\frac{W_{cond}-W_{dry}}{W_{dry}}\times 100\%$$

where *W_cond_* = weight of the conditioned sample at 20 °C, 65% RH, *W_dry_* = weight of the dry sample.

#### 2.5.2. Contact Angle Measurement

The wetting property of filter materials was examined by measuring water contact angle (CA) on filter surfaces, using a contact angle analyzer (SmartDrop Lab, Femtobiomed Inc, Seongnam, Korea). To measure the contact angle, water was used as a liquid. A fixed steel needle supplied a water drop of 3.0 ± 0.4 μL onto the surface of the fabric sample to be investigated. The image of the drops was captured. The data that resulted from processing the images used specific programs to fit the profile with the Young equation. At least five different points on each sample were considered.

#### 2.5.3. Measurement of Electric Potential

The electric field caused by the surface charges of filter media, or the electric potential of filter media, was measured using an electrostatic field meter (Simco-Model FMX-003, Simco ION, Hatfield, PA, USA). For measurement, the filter sample in 10 × 10 cm was hung in the air with a paper board placed behind the sample. The electric potential (static voltage) was measured from 2.54 cm away from the sample surface, and a total 64 measurements were done for a sample.

#### 2.5.4. Scanning Electron Microscopy

The morphology of sample was characterized using a field-emission scanning electron microscopy (FE-SEM, JSM-7800F, JEOL Ltd., Akishima, Tokyo, Japan), with prior Pt coating on fibrous samples for 70 s at 20 mA, using a sputter coater (108 auto, Cressington Scientific Inc., Watford, Hertfordshire, UK). The fiber diameter of all samples was measured by selecting 20 fibers randomly from SEM images.

#### 2.5.5. X-Ray Diffraction Analysis

The X-ray diffraction (XRD) of filter sample was analyzed using a powder X-ray diffractometer (SmartLab, Rigaku Corp., Tokyo, Japan). The obtained XRD data was used to calculate the change of crystallinity during heating by the XRD deconvolution method [29,30]. The deconvolution patterns of X-ray diffraction were analyzed using Gaussian function for each peak using ORIGIN PRO 8.5 software. The degree of crystallinity was calculated by the following in the range of 2θ = 10–30°.

(3) $$W_{C}=\frac{I_{C}}{I_{A}}\times 100\%$$

where *W_C_* = degree of crystallinity, *I_C_* = integrated area of crystalline peaks, *I_A_* = integrated area of all peaks.

#### 2.5.6. Analysis of Pore Size Distribution

A 1100-AEHXL capillary flow porometer (Porous Media Inc., Ithaca, NY, USA) was used to analyze the pore size distribution of filter webs. For measurement, the web samples in 3 × 3 cm was placed in the instrument, and soaked with the Galwick liquid (Porous Media Inc., Ithaca, NY, USA) having a low surface tension (15.9 dyn/cm). The air flow was blown to obtain wet and dry curves for analyzing the pore size distribution.

## 3. Results and Discussion

### 3.1. Electret Filter Materials

To examine the relationship between the charge retention of electrets and the electric conductivity of materials, the polymers with different dielectric constants (ε_r_) were chosen for this study; which were PP (ε_r_ ~ 2.2–2.6), PAN (ε_r_ ~ 4.2), and PVDF (ε_r_ ~ 8.4–8.9) [31,32,33]. PP was made into corona-charged meltblown filter media, and PAN and PVDF were made into electrospun filter media. For PP web, two different PP filter media with varied basis weight and thickness were used. Material properties and filter media characteristics are shown in Table 1.

**Figure 2:**
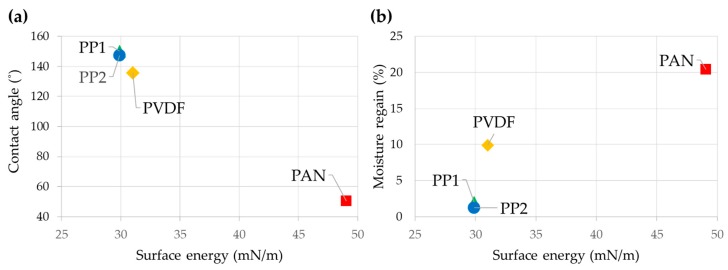
Materials’ affinity to water. (**a**) Contact angle and surface energy; (**b**) moisture regain and surface energy.

As the electric conductivity of material is also affected by material’s affinity to water [17], the wettability and moisture regain of materials were examined. Wettability, represented by the contact angle (CA) measurement, depends mostly on the surface energy of materials, if leaving the topography factor out. The surface energy of PP, PVDF, and PAN are 27.2–32.6, 25.5–36.5, and 44.0–54.1 mN/m, respectively [34,35,36,37,38]. The CAs of electret filter media are shown in Figure 2a, and a material with high surface energy showed a low CA, and vice versa. The moisture regain (%) measured at 20 °C, 65% RH was in the order of PAN (~ 20%) > PVDF (~ 10%) > PP (~ 2%), which were also in match with the order of surface energy (Figure 2b). The moisture regain of pure PP should be close to zero, but it was measured to be higher than the reference value [39], probably due to the additives in the meltblown web.

### 3.2. Effect of Environmental Conditions on the Charge Decay of Electret Filters

**Figure 3:**
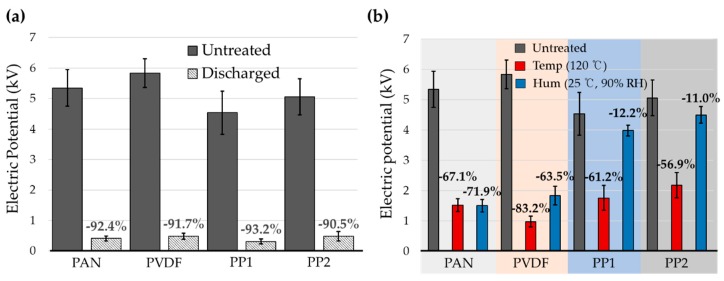
Electric potential measured for electret filters under various conditions. (**a**) Comparison of electric field from electret filter and discharged filter; (**b**) electric potential of electret filters before and after aging. Numerical values above the bar chart are the reduction (%) of electric potential for treatments, compared to the untreated filters.

To examine the charge decay phenomena of electret filters, the electric potential from each material was measured before and after the environmental aging. As aging factors, high temperature (120 °C, 90% RH), and high humidity (25 °C, 90% RH) were chosen, considering extreme environmental conditions that may apply during shelf-life or use. To make a reference of uncharged filter, the charges of the electret filter were intentionally killed by immersing the material in ethanol for 5 min. As shown in Figure 3a, over 90% of the electric potential was reduced after the ethanol-immersion, effectively discharging the filter.

After aging the electret filters at high temperature (temp) at 120 °C and at high humidity (hum) at 90% RH, respectively, the electric potential of filter decreased considerably. The reduction ratio of electric potential was varied for materials, and depending on aging conditions. The effect of thermal aging on the electret filter appeared largest for PVDF, followed by PAN, and then PP; which corresponded to the order of ε_r_ of materials. That is, materials with higher ε_r_ showed larger reduction of electric potential after aging at 120 °C. It is speculated that 120 °C was sufficiently high to mobilize the dipole and free charge carriers, causing the significant charge decay. The extent of charge decay seemed related to the electrical conductivity and thermal stability of charge carriers [42,43]. PVDF lost the electric potential as high as 83% of untreated filter, where PP lost about 55%~61% of the untreated.

The effect of moisture-aging on the electric potential was different for materials; the electric potentials of PAN, PVDF, and PP were reduced to 72%, 63.5%, and 11%–12%, respectively. The order of % reduction in electric potential corresponded to the order of surface energy of materials, where a material with higher surface energy and higher moisture regain showed more charge decay in the humid environment. Moisture absorbed in materials would facilitate the electrical conduction, thus the electret with higher moisture regain would be more vulnerable to humidity. Moreover, similar to thermal treatment, water molecules may influence the mobility of dipoles and charge carriers, acting as a plasticizer when absorbed in the polymer [17]. The results demonstrated that material parameters are involved in the extent of charge decay by thermal and humidity aging; a material with higher surface energy was more vulnerable to humidity in charge decaying, while the one with lower surface energy was less likely to be affected by moisture. With thermal aging, the ε_r_ of materials appeared to be involved with charge stability. PVDF as a polar material (with high ε_r_) cannot store space charges stably, while it can develop induced dipoles more strongly with polarization. Due to PVDF’s limited ability of charge storage, the electrostatic filtration performance could be easily deteriorated after thermal treatment. The effect of such aging on filtration performance was investigated in the following Section 3.3.

### 3.3. Effect of Aging Conditions on Filtration Performance

**Figure 4:**
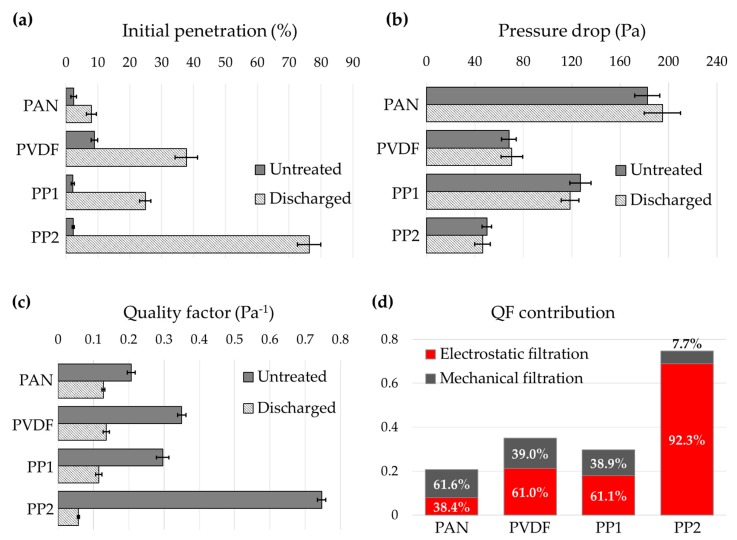
Filtration performance of untreated and discharged filter media. (**a**) Penetration of NaCl particles; (**b**) pressure drop at 12.5 cm/s; (**c**) quality factor; (**d**) contribution of mechanical vs. electrostatic filtration mechanisms.

The influence of charge decay on the filtration performance after aging was investigated in Figure 4. As performance parameters, % penetration of NaCl particles (count median diameter ~ 0.075 μm), pressure drop (Pa), and the quality factor (QF, Pa^−1^) were examined. QF is the relative filtration efficiency at a unit pressure drop, and is commonly used to evaluate the performance of filter at the same pressure drop.

From Figure 4, the NaCl penetrations of discharged filters increased considerably compared to those of untreated electret filters. The pressure drop of filter media changed a little after the solvent immersion; discharged PAN media increased the pressure drop by 6% because the fluffy structure was collapsed to a rather membrane-like structure after the solvent immersion, whereas the other media decreased the pressure drop in about 3%~7%. From the microscopic observation of the filter media (Figure S1), there are no apparent morphological changes after solvent immersion; the increase of penetration for the discharged media is mainly the result of charge loss, as supported by the measurement of electric potential in Figure 3a. As the fair comparison of filter performance of different filter media, the QF are compared in Figure 4c. While most of the filters lost more than 90% of electric potential, the reduction (%) of the QF were not as close as 90%, except the PP2 media; QF after discharging decreased by 38.4% for PAN, 61.0% for PVDF, 61.1% for PP1, and 92.3% for PP.

The reason that the charge loss was not fully reflected to the filter performance (QF) is the contribution from the mechanical filtration, in addition to the electrostatic filtration. Assuming that the performance of discharged filter is the result of mechanical filtration, the contribution percentages of the electrostatic filtration and the mechanical filtration were calculated in Figure 4d. PAN filter had the larger contribution from the mechanical particle capture mechanism, where PP2 showed the significantly larger contribution from the electrostatic filtration.

**Figure 5:**
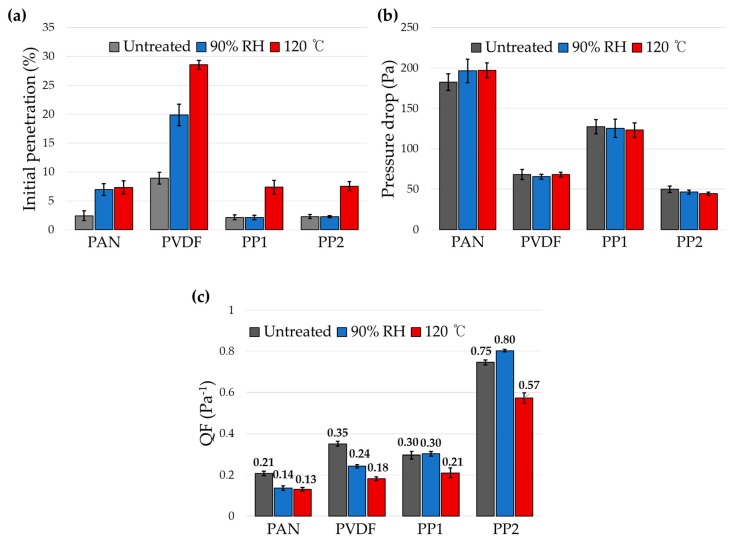
Effect of environmental aging on the filtration performance. (**a**) Penetration; (**b**) pressure drop; (**c**) quality factor.

From Figure 5, the effect of environmental humidity on filtration performance was largest for PAN media, followed by PVDF, and PP media. Due to the negligible moisture absorption of PP media, neither charges nor the QFs were affected by the moisture; on the contrary, the QF of PP media appeared to increase after humidity aging, due to the slight decrease of pressure drop after aging (Figure 5c). Regarding the pressure drop change after aging, there were no specific trends of pore size changes observed after thermal and humidity aging, from the analysis of pore size distribution in Figure S2.

**Figure 6:**
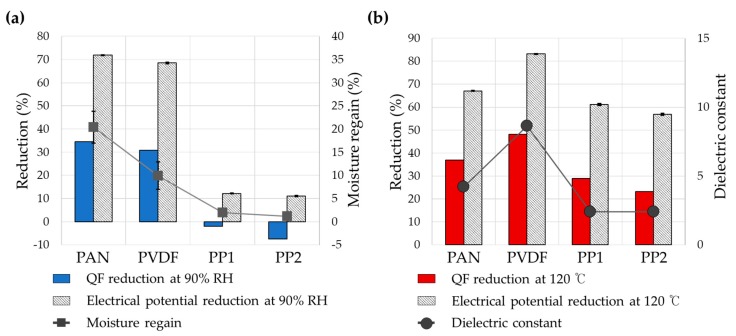
Effect of humidity and temperature on the electric potential and quality factor (QF). (**a**) Material’s moisture regain vs. the reduction of QF and electric potential with humidity aging; (**b**) material’s dielectric constant vs. the reduction of QF and electric potential with thermal aging.

The largest extent of QF decrease occurred in PAN, of which moisture regain was highest among the tested. The penetration of PAN and PVDF increased by 2.9 and 1.6 times, respectively, by aging at 90% RH. As a result, QF decreased by 34.5% and 30.8% for PAN and PVDF, respectively. The level of moisture regains well agreed with the extent of charge decay and the performance reduction (%) after humidity aging (Figure 6a). The effect of thermal aging on the filtration performance appeared significantly large for all filter samples (Figure 6b). The % decrease of QF by aging at 120 °C were 36.8% for PAN, 48.1% for PVDF, 29.0% for PP1, and 23.3% for PP2, and the order of QF reduction corresponded to the order of ε_r_ of polymeric materials.

Generally, the dielectric property of a material is affected by the electron hopping at each energy level, where the band gap energy becomes important [14,15,44]. From Equation (4) [15,44], the dielectric constant is inversely proportional to the square of the band gap energy. The material with a higher ε_r_ would have a smaller band gap energy, and it can be predicted that the loss of charge is much easier in materials with high ε_r_. Similarly, in Equation (5) [14], the electrical conductivity can be expressed by the band gap energy; which shows that the material with high electrical conductivity would have a smaller band gap.

(4) $$\varepsilon_{r}=1+{\left(\frac{1460\;\mathrm{kJ}/\mathrm{mol}}{E_{g}}\right)}^{2}$$

where $\varepsilon_{r}$ = relative dielectric constant, $E_{g}$ = band gap energy.

(5) $$\mu \left(T\right)\approx \mu_{0}\left(T\right)\exp\left(-\frac{E_{g}}{2RT}\right)$$

where μ(T) = electrical conductivity at temperature T (K), $\mu_{0}$(T) = pre-exponential constant at temperature T (K), R = gas constant (8.314 J/mol∙K), T = temperature (K), $E_{g}$ = band gap energy (kJ/mol).

### 3.4. Effect of Crystallinity on Charge Decay with Thermal Aging

**Figure 7:**
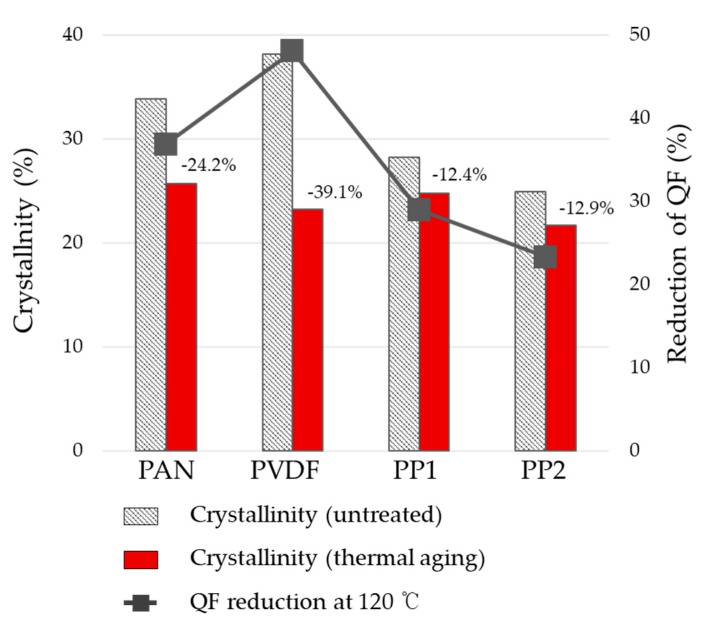
Relationship of crystallinity reduction (%) and QF reduction (%) with thermal aging. The percentage values above the bar charts are the reduction % of crystallinity with thermal aging.

The effect of material crystallinity on charge decay has not been clarified so far. Zhang et al. [45,46] reported that the degree of crystallinity is hardly associated with charge decay. It is also reported that a material with high crystallinity would have a short distance for charge carriers, thus giving a high electrical conductivity [47,48]. To examine the effect of crystallinity on charge decay, the crystallinities (X-tallinity) of polymers before and after thermal aging were analyzed, and their influences on the filtration performances (QF) were investigated (Table 2 and Figure 7). The crystallinity (%) of polymer was measured by the X-ray diffraction analysis (XRD); the fittings of crystalline curve and the amorphous curve are detailed in Figure S3.

For all polymers, the crystallinity decreased after the thermal aging. PVDF, which had the highest crystallinity at the beginning, showed the largest extent of decrease in the crystallinity, whereas the PP with the lowest crystallinity originally showed the smallest extent of decrease in the crystallinity with thermal aging. The reduction of crystallinity with thermal aging corresponded with the extent of QF reduction; also, the material with the highest crystallinity, PVDF, displayed the largest impact on the charge decay and filtration performance after thermal aging. Although it is argumentative, the result agrees with the speculation that the material with a high crystallinity tends to lose charges more easily.

The results of this study implicate that dielectric (ε_r_) and wetting properties are important parameters influencing the electric conductivity and chain mobility, and they can be used as convenient predictors for the charge retention capacity of electret filter materials.

## 4. Conclusions

Electret filters as opposed to mechanical filters have the benefit of electrostatic filtration, but this benefit can be limited when the electret charges are deteriorated by the environmental aging. The objective of this work is to investigate the polymeric parameters that influence the charge retention capacity of electret filters. Electrets with three different dielectric constants—PP, PAN, PVDF—were compared for their charge decay phenomena, and their impacts on the electrostatic filtration performances were investigated, with the accelerated aging conditions under high temperature (120 °C) or high humidity (25 °C, 90% RH) for 48 h. The effect of moisture-aging on charge decay was highest in the order of PAN > PVDF > PP, corresponding to the order of surface energy. The deterioration of filtration performance, in terms of QF, also agreed with the charge decay. The effect of thermal aging on charge decay appeared largest for PVDF that has the highest ε_r_, followed by PAN and then PP. With thermal aging, the material with a high ε_r_ was more vulnerable to charge loss, leading to the deterioration of filtration performance. The effect of thermal aging on the filtration performance appeared significantly large than the effect of humidity aging for all filter samples. For all polymers, the crystallinity decreased after the thermal aging. PVDF, which had the highest crystallinity, showed the largest extent of decrease in crystallinity. Moreover, the material with the highest crystallinity, PVDF, showed the largest impact on charge decay and filtration performance with thermal aging.

Ultimately, this study intends to provide information on the polymeric attributes that affect the charge retention capability, for design of robust filter material of which charges sustain in harsh environmental conditions. The results of this study implicate that ε_r_ and wettability are important parameters influencing the electric conductivity and chain mobility, and they can be used as a convenient predictor for the charge retention capacity. This study will broadly impact the development of robust filters, by guiding the material selection for the superior electrostatic filtration.

## Figures and Tables

**Table 1:** Material properties and filter media characteristics.

	PAN	PVDF	PP1	PP2
Surface Energy (mJ/m^2^) [34,35,36,37,38,39]	44.0 ~ 54.1	25.5 ~ 36.5	27.2 ~ 32.6	
ε_r_ (100 Hz, 1 MHz) [31,32]	4.2	8.4 ~ 8.9	2.2 ~ 2.6	
T_g_ (°C) [40]	95.5 ~ 105	−50 ~ −20	−20 ~ −10	
T_m_ (°C) [41]	300 ~ 320	~ 175	~ 173	
Web type	electrospun	electrospun	meltblown	
Weight (g/m^2^) (n = 5)	2.4 (± 0.26)	5.1 ( ± 0.28)	47.3 (± 1.01)	28.9 (± 0.64)
Thickness (mm) (n = 5)	0.28 (± 0.02)	0.30 ( ± 0.03)	0.34 (± 0.02)	0.29 (± 0.02)
Fiber diameter (μm) (n = 30)	0.55 (± 0.13)	1.01 (± 0.36)	2.49 (± 0.87)	3.44 (± 1.71)

**Notation.** T_g_: Glass transition temperature; T_m_: Melting temperature; the surface energy: ε_r_, T_g_, and T_m_ are reference values [31,32,34,35,36,37,38,39,40,41]; weight: Thickness and effective fiber diameter are measured values; ‘n’ is the number of samples tested or measured.

**Table 2:** Effect of thermal aging on the crystallinity and QF.

	X-Tallinity Before Aging (%)	QF Before Aging (Pa^−1^)	X-Tallinity After Aging (%)	QF After Aging (Pa^−1^)	X-Tallinity Reduction After Aging (%)	QF Reduction After Aging (%)
PAN	33.87	0.21	25.68	0.13	24.2	36.9
PVDF	38.21	0.35	39.08	0.18	39.1	48.1
PP1	28.28	0.30	12.39	0.21	12.4	29.0
PP2	24.90	0.75	12.87	0.57	12.9	23.3

## Supplementary Materials

The following are available online at https://www.mdpi.com/2073-4360/12/3/721/s1.
