# Supplementary material for: Material Properties Influencing the Charge Decay of Electret Filters and their Impact on Filtration Performance
Source: Polymers (Basel). 2020 Mar 24;12(3):721. doi: 10.3390/polym12030721 (PMC7183080; doi:10.3390/polym12030721)
Supplement: Supplementary file 1 [file polymers-12-00721-s001.pdf]

Supplementary Materials:

Material properties influencing the charge decay of electret filters and their impact on filtration performance

Jinwook Lee [1] and Jooyoun Kim [1,2,*]

[1] Department of Textiles, Merchandising and Fashion Design, Seoul National University, Seoul 08826, Korea; shop0319@snu.ac.kr
[2] Research Institute of Human Ecology, Seoul National University; jkim256@snu.ac.kr

* Correspondence: jkim256@snu.ac.kr; Tel.: +82-2-880-6846

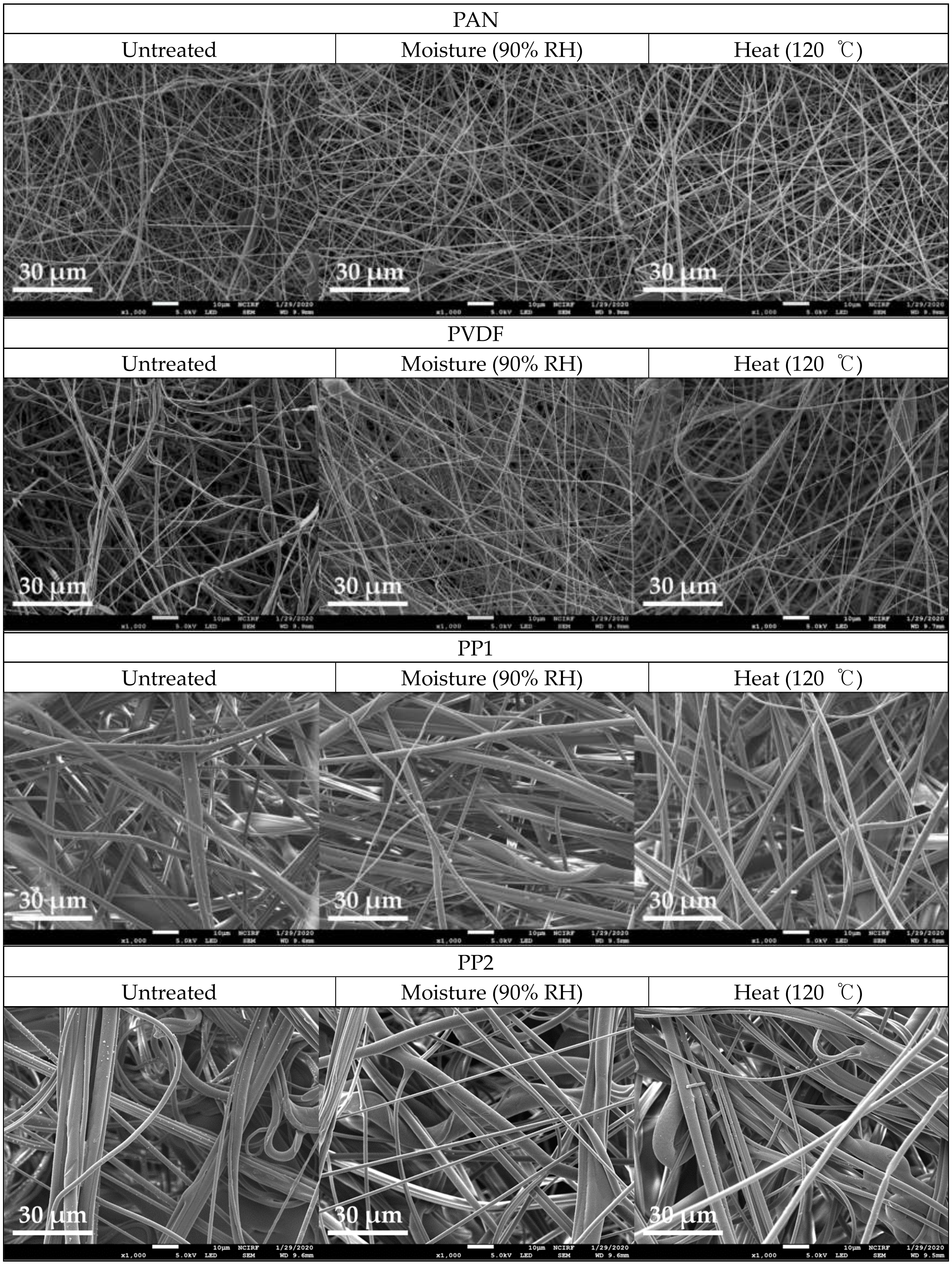

Figure S1. Scanning electron microscope (SEM) images of filter webs with different aging conditions.

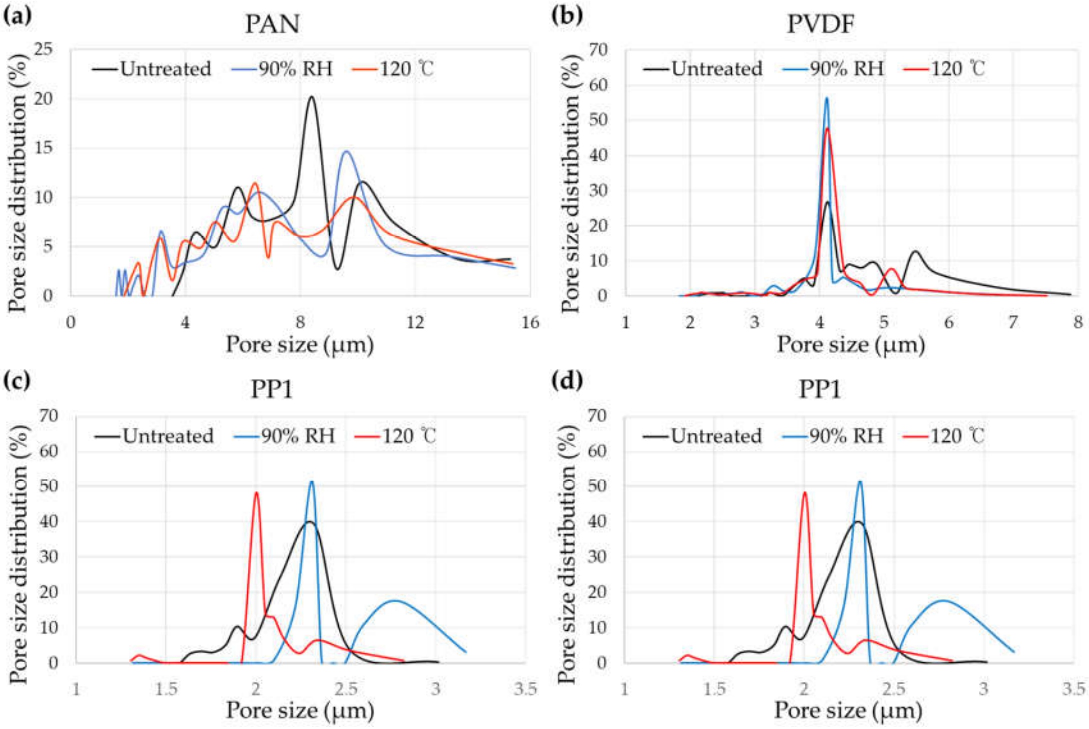

Figure S2. Pore size distribution of filter webs.

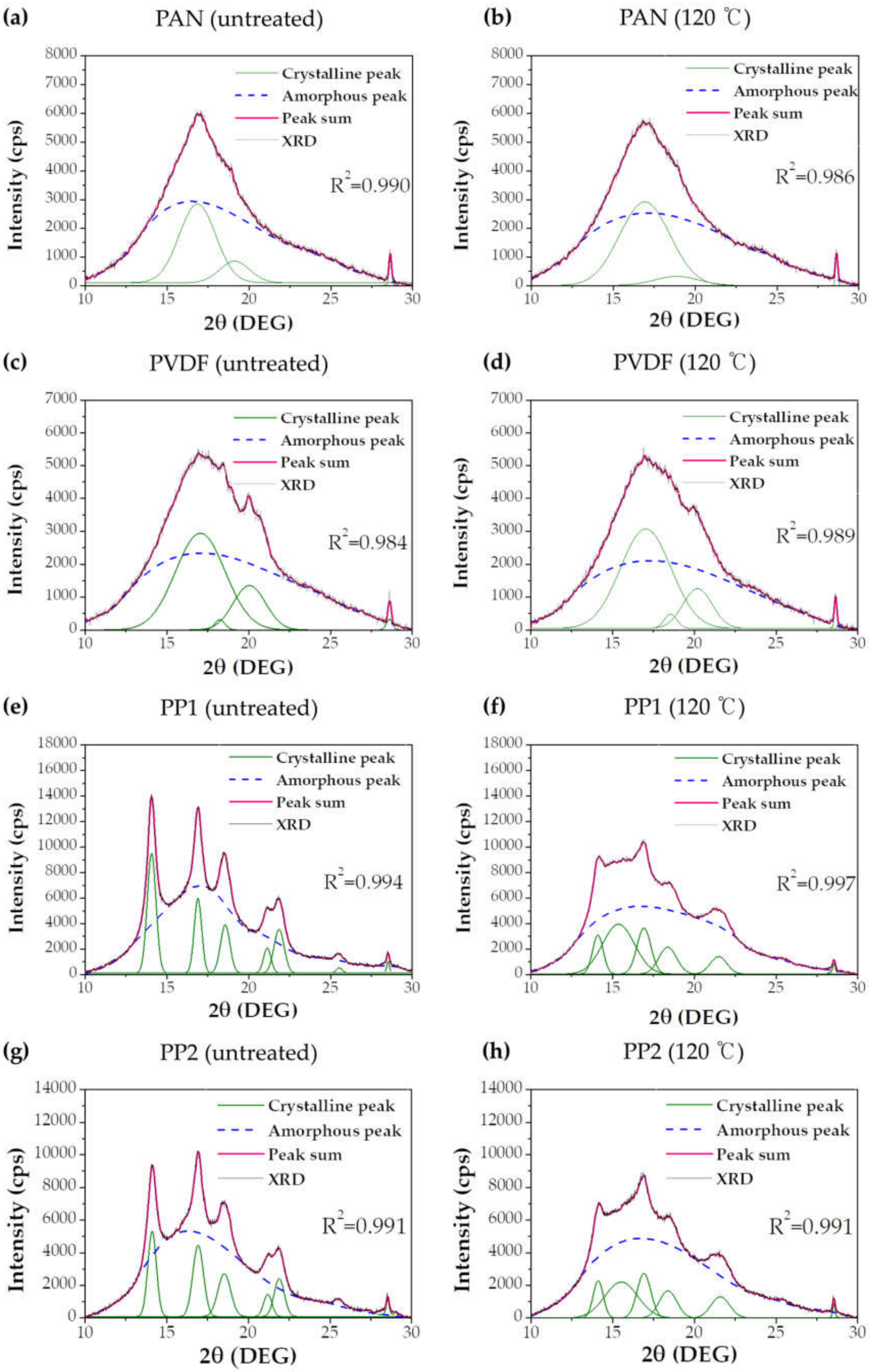

Figure S3. XRD patterns of untreated and heat treated filter sebs.
